# Smoking interacts with *HLA-DRB1 *shared epitope in the development of anti-citrullinated protein antibody-positive rheumatoid arthritis: results from the Malaysian Epidemiological Investigation of Rheumatoid Arthritis (MyEIRA)

**DOI:** 10.1186/ar3813

**Published:** 2012-04-26

**Authors:** Chun Lai Too, Abqariyah Yahya, Shahnaz Murad, Jasbir Singh Dhaliwal, Per Tobias Larsson, Nor Asiah Muhamad, Nor Aini Abdullah, Amal Nasir Mustafa, Lars Klareskog, Lars Alfredsson, Leonid Padyukov, Camilla Bengtsson

**Affiliations:** 1 Rheumatology Unit, Department of Medicine, Center of Molecular Medicine L8:04, Karolinska University Hospital, 17176 Stockholm, Sweden; 2 Institute for Medical Research, Jalan Pahang, 50588 Kuala Lumpur, Malaysia; 3 Institute of Environmental Medicine, Karolinska Institutet, Nobel väg 13, Box 210, 17177 Stockholm, Sweden

## Abstract

**Introduction:**

Rheumatoid arthritis (RA) is a multifactorial autoimmune disease in which genetic and environmental factors interact in the etiology. In this study, we investigated whether smoking and *HLA-DRB1 *shared-epitope (SE) alleles interact differently in the development of the two major subgroups of rheumatoid arthritis (RA), anti-citrullinated proteins antibody (ACPA)-positive and ACPA-negative disease, in a multiethnic population of Asian descent.

**Methods:**

A case-control study comprising early diagnosed RA cases was carried out in Malaysia between 2005 and 2009. In total, 1,076 cases and 1,612 matched controls participated in the study. High-resolution *HLA-DRB1 *genotyping was performed for shared-epitope (SE) alleles. All participants answered a questionnaire on a broad range of issues, including smoking habits. The odds ratio (OR) of developing ACPA-positive and ACPA-negative disease was calculated for smoking and the presence of any SE alleles separately. Potential interaction between smoking history (defined as "ever" and "never" smoking) and *HLA-DRB1 *SE alleles also was calculated.

**Results:**

In our multiethnic study, both the SE alleles and smoking were associated with an increased risk of developing ACPA-positive RA (OR SE alleles, 4.7; 95% confidence interval (CI), 3.6 to 6.2; OR smoking, 4.1; 95% CI, 1.9 to 9.2). SE-positive smokers had an odds ratio of ACPA-positive RA of 25.6 (95% CI, 10.4 to 63.4), compared with SE-negative never-smokers. The interaction between smoking and SE alleles was significant (attributable proportion due to interaction (AP) was 0.7 (95% CI, 0.5 to 1.0)). The HLA-DRB1*04:05 SE allele, which is common in Asian populations, but not among Caucasians, was associated with an increased risk of ACPA-positive RA, and this allele also showed signs of interaction with smoking (AP, 0.4; 95% CI, -0.1 to 0.9). Neither smoking nor SE alleles nor their combination was associated with an increased risk of ACPA-negative RA.

**Conclusions:**

The risk of developing ACPA-positive RA is associated with a strong gene-environment interaction between smoking and *HLA-DRB1 *SE alleles in a Malaysian multiethnic population of Asian descent. This interaction seems to apply also between smoking and the specific HLA-DRB1*04:05 SE allele, which is common in Asian populations but not in Caucasians.

## Introduction

Rheumatoid arthritis (RA) is a multifactorial autoimmune disease in which genetic and environmental factors interact in the etiology of the disease [1,2]. The strongest gene association is considered to be within the human leukocyte antigen (HLA) region, particularly the *HLA-DRB1 *genes. Multiple *HLA-DRB1 *alleles encoding the shared epitope (SE) at amino acid positions 70 to 74 in the third hypervariable region of the *DRβ1 *molecules are associated with a higher risk for RA [3].

Relatively little is known about environmental factors that may contribute to the disease, except smoking, which is the main environmental factor that has consistently been related to an increased risk of RA [4-11]. Recent data revealed a strong gene-environment interaction between smoking and SE alleles in the risk of ACPA-positive RA. However, the majority of studies undertaken so far have focused on the Caucasian populations, with consistent findings [5-8,10-13]. In contrast, a cross-sectional study of African Americans with early RA did not find an association between smoking and ACPA-positive RA [14]. A study of an Asian population on prevalent RA cases demonstrated that the combination of SE alleles and smoking was associated with risk of RA, regardless of ACPA status [4]. A gene-environment interaction between smoking and SE alleles was, however, observed only for ACPA-positive RA in this study.

In the Malaysian Epidemiological Investigation of Rheumatoid Arthritis (MyEIRA) case-control study, we recently reported that *HLA-DRB1 SE *alleles were consistently associated with ACPA-positive RA in three Asian ethnic populations [15]. Furthermore, our group also demonstrated that smoking is associated with RA development and that this effect is restricted to the ACPA-positive RA subset [16]. However, the question whether the *HLA-DRB1 *SE alleles and smoking interacts in providing an increased risks for ACPA-positive RA remains to be answered in our non-Caucasian population. This question is of particular relevance, as the occurrence of different *HLA-DRB1 *SE alleles is different between Asian and Caucasian populations.

In the present study, we used the first large Asian case-control study performed in Malaysia involving early RA to investigate the interaction between smoking and *HLA-DRB1 *SE alleles (including those that are rare in Caucasian populations) regarding risk of disease.

## Materials and methods

### Study design

The source of data for our investigation was the MyEIRA case-control study, in which the subjects were individuals aged 18 to 70 years in Peninsular Malaysia. The recruitment period for cases and controls was between August 2005 and December 2009.

### Identification of cases and controls

The details of the MyEIRA study have been described elsewhere [16]. In brief, patients with early RA were identified from nine centers throughout Peninsular Malaysia. All cases were diagnosed by rheumatologists according to the 1987 revised American College of Rheumatology criteria [17]. The clinical and radiographic data were recorded in a binary fashion (present or absent). For each case, a population control was randomly selected matched by age, sex, and residential area. In addition, hospital-based controls were recruited in the first 2 years of the study period. These were selected among hospital staff, people who accompanied patients, and patients without any underlying autoimmune diseases. Participation in the study was voluntary, and if an eligible control decided not to participate, another control was selected by using the same procedure. Written consent was obtained from all participants. The Medical Research and Ethnics Committee, Ministry of Health, Malaysia, approved the study.

### Data collection and blood sampling

Information about environmental exposures was collected from cases and controls by means of an identical questionnaire used in a face-to-face interview by well-trained interviewers. Cases and controls were also asked to provide a blood sample. Of the 1,166 identified early RA cases, 1,076 (92%) completed a questionnaire. In total, 2,112 controls were identified in the study, and the participation proportion was 76%, resulting in 1,612 controls available for analysis. Sera for serologic analysis and cells for DNA preparation were obtained from all the participating subjects.

### DNA extraction and *HLA-DRB1 *genotyping

White cells were separated by using Ficoll Hypaque, and the DNA was extracted by using the QIAamp DNA Blood Mini Kit from Qiagen (Hilden, Germany). All DNA was stored at -20°C until tested. *HLA-DRB1 *two-digit and 4-digit genotyping were performed by the PCR-SSO method described elsewhere [15]. The overall genotyping success rate was 99.9%. DNA samples from two RA cases were of poor quality in genotyping and therefore excluded from the statistical evaluation. The *HLA-DRB1 *shared epitope (SE) alleles group was defined by HLA-DRB1*01:01, *01:02, *01:07, *04:01, *04:04, *04:05, *04:08, *04:10, *10:01, and *10:03. More than 95% of the HLA-DRB1*10 subtypes were HLA-DRB1*10:01. A preponderance of *HLA-DRB1 *SE alleles occurred in the RA cases compared with controls (40% versus 16%). Only 46 (4.4%) of 1,054 RA patients carried two copies of *HLA-DRB1 *SE alleles. Thus, we decided to limit the analysis to investigate the influence from any SE allele (either one or two copies of SE alleles) on RA risk.

### Autoantibody measurements

Anti-citrullinated peptide antibody (ACPA) was quantified by using the anti-CCP ELISA kits (Immunoscan-RA, Malmö, Sweden) for all subjects [15]. An antibody level ≥25 AU/ml was regarded as ACPA positive. For the determination of IgM and IgG rheumatoid factors, we used the ELISA kits of Immuno-Biological Laboratories, Hamburg, Germany. An antibody level > 15 IU/ml was defined as positive.

### Definition of smoking status

Cases and controls were classified as "ever-smokers" and "never-smokers," according to their reported smoking habits. In brief, ever-smokers were defined as those who smoked before or during the index year (the time point at which RA symptoms started and the corresponding time point among the matched controls), as described previously [16]. Never-smokers are those who had never smoked before or during the index year.

### Statistical analysis

In the analyses, data from cases and their matched controls were used. Twenty cases and 196 controls lacked information from a matched control/case, and therefore in total, 1,056 cases and 1,416 controls (1,056 matched population controls and 360 matched hospital controls) were retained in the analyses. Smoking history was obtained from 1,006 patients with RA (95.3%) and from 1,325 control subjects (93.6%). Of these, 79 of the cases had ever smoked (70 men and nine women), and 65 of the control subjects were ever-smokers (60 men and five women) (Table 1).

**Table 1:** Characteristics of rheumatoid arthritis cases and controls in the MyEIRA study

	All		Men		Women	
	
	Cases *n *= 1,056	Controls *n *= 1,416	Cases *n *= 151	Controls *n *= 211	Cases *n *= 905	Controls *n *= 1,205
Age, mean (SD) years	47.7 (11.5)	47.1 (11.4)	50.1 (11.9)	49.8 (11.9)	47.3 (11.4)	46.7 (11.3)
Smoking status						
Never-smoker, *n *(%)	927 (88)	1,260 (89)	61 (40)	101 (48)	866 (96)	1159 (96.2)
Ever-smoker, *n *(%)	79 (7)	65 (5)	70 (46)	60 (28)	9 (1)	5 (0.4)
Information missing on smoking, *n *(%)	50 (5)	91 (6)	20 (14)	50 (24)	30 (3)	41 (3.4)
Ethnic group						
Malay	441 (42)	893 (63)	66 (44)	126 (60)	375 (41)	767 (64)
Chinese	217 (20)	183 (13)	36 (24)	31 (15)	181 (20)	152 (13)
Indian	323 (31)	253 (18)	37 (25)	43 (20)	286 (32)	210 (17)
Others	75 (7)	87 (6)	12 (8)	11 (5)	63 (7)	76 (6)
ACPA status						
ACPA-positive RA, *n *(%)	680 (64)	32 (2)	104 (69)	5 (2)	576 (64)	27 (2)
ACPA-negative RA, *n *(%)	376 (36)	1,383 (98)	47 (31)	206 (98)	329 (36)	1,177 (98)
Presence of SE alleles						
None SE alleles, *n *(%)	629 (60)	1,183 (84)	83 (55)	181 (86)	546 (60)	1,002 (83)
Any SE alleles, *n *(%)	425 (40)	233 (16)	68 (45)	30 (14)	357 (39)	203 (17)

ACPA, anti-citrullinated peptide antibody; DNA, deoxyribonucleic acid; MyEIRA, Malaysian Epidemiological Investigation of rheumatoid arthritis; RA, rheumatoid arthritis; SD, standard deviation; SE, shared epitope. The "any SE alleles" group was defined by the presence of either one or two HLA-DRB1*01:01, *01:02, *01:07, *04:01, *04:04, *04:05, *04:08, *04:10, *10:01, and/or *10:03 SE alleles.

The odds ratio (OR) and 95% confidence interval (95% CI) of developing ACPA-positive RA, ACPA-negative RA, and RA in total, associated with smoking habits and the presence of SE alleles, were calculated by using conditional logistic regression. Never-smokers without SE-alleles were used as reference group. HLA-DRB1*04:05 is the most common allele within the HLA-DRB1*04 group in our Malaysian population (the allelic frequency was 15.5% among cases and 5.7% among controls). Subsequently, we performed additional analyses by stratifying the SE-positive individuals into two groups: the HLA-DRB1*04:05 group and the non-HLA-DRB1*04:05 group (defined by HLA-DRB1*01:01, *01:02, *01:07, *04:01, *04:04, *04:08, *04:10, *10:01, and *10:03). Cases (*n *= 13) and controls (*n *= 1) carrying HLA-DRB1*04:05 as first allele and a non-HLA-DRB1*04:05 SE as second allele were not included in this analysis.

Interaction between smoking habits and the presence of SE alleles was evaluated as a departure from the additivity of effects and was estimated by calculating the attributable proportion due to interaction (AP) together with the 95% CI [18].

Potential confounding factors (that is, formal educational level (categorized as no formal education, primary education, secondary education or college/university)) and ethnicity (categorized as Malay, Chinese, Indian, and Others), were adjusted for in all analyses. The SAS software, version 9.2 (SAS Institute, Cary, NC, USA) was used for computations.

## Results

### Characteristics of the subjects

In this report, the 2,110 participants (*n *= 905 RA cases and 1,205 controls) were women, and 362 were men (*n *= 151 RA cases and *n *= 211 controls). The median duration of time from disease onset to inclusion in the study was 1 year, with an interquartile range (IQR) of 2 years for both men and women. Radiographic data were recorded as presence or absence of hand-joint erosions. In total, 974 radiographic erosion data were available in the present study. Of these, 169 (17.4%) of 974 patients had erosive RA. Extraarticular features were present in 114 (11.7%) patients with RA: 52 (5.3%) patients had sicca syndrome; 28 (2.9%) patients had lung fibrosis; 19 (2.0%) patients had rheumatoid nodules; 14 (1.4%) patients had secondary Sjögren syndrome; and one patient had vasculitis. Ever-smoking was more frequent in men for both cases (48%) and controls (28%), as compared with women (1% among cases and 0.4% among controls). In total, 64% of the cases were ACPA positive, and 40% of the cases were carriers of SE alleles. Of the 680 ACPA-positive RA cases, 344 (51%) individuals carried SE alleles; 51.2% and 51.7% of RA patients were IgM RF-positive and IgG RF-positive, respectively. The distribution of ethnic groups (Malays, Chinese, Indians, and other subethnicities) is presented in Table 1. Men were slightly older than women. The proportion with higher education was higher among the controls than the cases (74% versus 66%, data not shown).

### Smoking and the risk of ACPA-positive/-negative RA

The odds ratio of developing ACPA-positive RA was 4.1 (95% CI, 1.9 to 9.2), among ever-smokers compared with never-smokers (Table 2). This increased risk was seen in both men and women, but the number of smokers among women was small. No association was found between smoking and the risk of developing ACPA-negative RA (OR = 0.7; 95% CI, 0.3 to 2.0).

**Table 2:** Odds ratios of developing RA overall, ACPA-positive RA, and ACPA-negative RA among ever-smokers compared with never-smokers in MyEIRA

	**All**		**Men**		**Women**	
	
	**Ca/Co**	**OR (95% CI)**	**Ca/Co**	**OR (95% CI)**	**Ca/Co**	**OR (95% CI)**
	
All RA						
Never-smokers	927/1,260	1.0	61/101	1.0	866/1,159	1.0
Ever-smokers	79/65	2.2 (1.2 to 3.9)	70/60	2.0 (1.0 to 4.0)	9/5	1.9 (0.9 to 6.1)
ACPA-positive RA						
Never-smokers	577/1,260	1.0	33/101	1.0	544/1,159	1.0
Ever-smokers	64/65	4.1 (1.9 to 9.2)	56/60	3.5 (1.3 to 9.4)	8/5	3.6 (0.7 to 18.4)
ACPA-negative RA						
Never-smokers	350/1,260	1.0	28/101	1.0	322/1,159	1.0
Ever-smokers	15/65	0.7 (0.3 to 2.0)	14/60	0.8 (0.2 to 3.4)	1/5	0.5 (0.05 to 4.7)

ACPA, anti-cyclic citrullinated proteins antibody; Ca/Co is number of exposed cases/number of exposed controls; MyEIRA, Malaysian Epidemiological Investigation of Rheumatoid Arthritis; OR (95% CI), odds ratio and 95% confidence interval, adjusted for ethnicity and formal education by using conditional logistic regression; RA, rheumatoid arthritis.

### *HLA-DRB1 *SE alleles and the risk of ACPA-positive/negative RA

Carriers of *HLA-DRB1 *SE alleles had an increased risk of developing ACPA-positive RA (OR = 4.7; 95% CI, 3.6 to 6.2), compared with those without SE alleles (Table 3). When the HLA-DRB1*04:05 allele group was analyzed separately, this subgroup also showed an association with an increased risk of ACPA-positive RA (OR = 6.5; 95% CI, 4.9 to 8.7). No increased risk of ACPA-negative RA was found in individuals carrying any SE allele (OR = 1.2; 95% CI, 0.8 to 1.7), or the HLA-DRB1*04:05 allele when this was investigated separately (OR = 1.2; 95% CI, 0.8 to 1.9).

**Table 3:** Odds ratios of developing RA overall, ACPA-positive RA, or ACPA-negative RA among carriers of SE alleles compared with individuals without SE alleles in MyEIRA

	All		Men		Women	
	
	Ca/Co	OR (95% CI)	Ca/Co	OR (95% CI)	Ca/Co	OR (95% CI)
All RA						
No SE allele	629/1,183	1.0	83/181	1.0	546/1,002	1.0
Any SE alleles	425/233	3.0 (2.4 to 3.7)	68/30	4.5 (2.6 to 7.8)	357/203	2.8 (2.3 to 3.5)
ACPA-positive RA						
No SE allele	334/1,183	1.0	45/181	1.0	289/1,002	1.0
Any SE alleles	345/233	4.7 (3.6 to 6.2)	59/30	8.0 (3.7 to 17.2)	286/203	4.3 (3.2 to 5.7)
HLA-DRB1*04:05	158/86	6.5 (4.9 to 8.7)	29/16	7.3 (3.3 to 14.6)	129/70	6.4 (4.6 to 8.8)
Non-HLA-DRB1*04:05 SE alleles	187/147	4.5 (3.5 to 5.8)	30/14	8.6 (4.2 to 17.6)	157/133	4.1 (3.1 to 5.3)
**ACPA-negative RA**						
No SE allele	295/1,183	1.0	38/181	1.0	257/1,002	1.0
Any SE alleles	80/233	1.2 (0.8 to 1.7)	9/30	1.5 (0.6 to 3.9)	71/203	1.2 (0.8 to 1.7
HLA-DRB1*04:05	26/86	1.2 (0.8 to 1.9)	4/16	1.2 (0.4 to 3.8)	22/70	1.2 (0.7 to 2.0)
Non-HLA-DRB1*04:05 SE alleles	54/147	1.5 (1.1 to 2.1)	5/14	1.7 (0.6 to 5.0)	49/133	1.4 (1.0 to 2.0)

ACPA, anti-cyclic citrullinated proteins antibody; Ca/Co, the number of exposed cases/number of exposed controls; MyEIRA, Malaysian Epidemiological Investigation of Rheumatoid Arthritis; OR (95% CI), odds ratio and 95% confidence interval, adjusted for ethnicity and formal education by using conditional logistic regression; RA, rheumatoid arthritis. The "any SE alleles group" was defined by the presence of either one or two HLA-DRB1*01:01, *01:02, *01:07, *04:01, *04:04, *04:05, *04:08, *04:10, *10:01, and/or *10:03. The "HLA-DRB1*04:05 group" was defined by the presence of either one or two HLA-DRB1*04:05 allele.

### Interaction between smoking and SE alleles in relation to ACPA-positive/-negative RA

**Figure 1:**
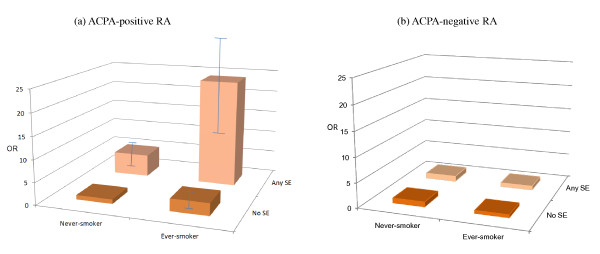
**Odds ratios of developing ACPA-positive and ACPA-negative rheumatoid arthritis (RA) for different combinations of smoking and SE alleles**. The odds ratios of developing **(a) **ACPA-positive RA and **(b) **ACPA-negative RA were compared with those who never smoked and have no SE alleles.

We observed an increased risk of ACPA-positive RA among ever-smokers without SE alleles (OR = 3.1; 95% CI, 1.7 to 5.7), compared with never-smokers without SE alleles (Table 4). In never-smokers with SE alleles, the OR for ACPA-positive RA was 4.4 (95% CI, 3.5 to 5.6); compared with never-smokers without the SE allele. A dramatically increased risk for ACPA-positive RA was seen in individuals who were both smokers and *HLA-DRB1 *SE positive (OR = 25.6; 95% CI, 10.4 to 63.4), and here we also observed a statistically significant gene-environment interaction between smoking and the *HLA-DRB1 *SE (AP = 0.7; 95% CI, 0.5 to 1.0; Figure 1). The combination of smoking and the HLA-DRB1*04:05 SE allele was also associated with an increased risk of ACPA-positive RA (OR = 12.9; 95% CI, 4.7 to 35.3; Table 5), and signs of interaction were seen, albeit statistically insignificant (AP = 0.4; 95% CI, -0.1 to 0.9).

**Table 4:** Odds ratios of developing ACPA-positive RA and ACPA-negative RA in subjects exposed to different combinations of smoking and SE alleles compared with never-smokers without SE alleles in MyEIRA

	**No SE**		**Any SE**	
	
	**Ca/Co**	OR (95% CI)*	**Ca/Co**	OR (95% CI)*
	
ACPA-positive RA				
Never-smokers	266/986	1.0	264/202	4.4 (3.5 to 5.6)
Ever-smokers	30/52	3.1 (1.7 to 5.7)	33/8	25.6 (10.4 to 63.4)
AP				0.7 (0.5 to 1.0)
				
ACPA-negative RA				
Never-smokers	254/986	1.0	65/202	1.1 (0.7 to 1.7)
Ever-smokers	10/52	0.6 (0.2 to 2.0)	3/8	0.8 (0.1 to 4.8)

ACPA, anti-cyclic citrullinated proteins antibody; AP, attributable proportion due to interaction; Ca/Co, number of exposed cases/number of exposed controls; MyEIRA, Malaysian Epidemiological Investigation of Rheumatoid Arthritis; OR (95% CI), odds ratio and 95% confidence interval, adjusted for ethnicity, formal education, age, sex, residential area, *calculated by means of unconditional logistic regression to increase power; RA, rheumatoid arthritis; SE, shared epitope.

**Table 5:** Odds ratios for developing ACPA-positive RA and ACPA-negative RA in subjects exposed to different combinations of smoking and presence of HLA-DRB1*04:05 or non-HLA-DRB1*04:05 SE alleles, compared with never-smokers without SE alleles in MyEIRA

	No SE alleles		HLA-DRB1*04:05 SE alleles^**ab**^		Any non-HLA-DRB1*04:05 SE alleles^**c**^	
	
	Ca/Co	OR (95% CI)	Ca/Co	OR (95% CI)	Ca/Co	OR (95% CI)
ACPA-positive RA						
Never-smokers	266/986	1.0	102/71	5.4 (3.8 to 7.5)	152/130	3.1 (2.4 to 4.0)
Ever-smokers	30/52	3.1 (1.7 to 5.7)	16/7	12.9 (4.7 to 35.3)	15/1	82.4 (10.3 to 656.9)
AP				0.4 (-0.1 to 0.9)		0.9 (0.8 to 1.1)
						
ACPA-negative RA						
Never-smokers	254/986	1.0	20/71	1.1 (0.7 to 1.8)	44/130	1.3 (0.9 to 1.9)
Ever-smokers	10/52	0.6 (0.2 to 2.0)	1/7	0.6 (0.1 to 4.8)	2/1	8.1 (0.7 to 93.0)

ACPA, anti-cyclic citrullinated proteins antibody; AP, attributable proportion due to interaction; Ca/Co, number of exposed cases/number of exposed controls; MyEIRA, Malaysian Epidemiological Investigation of Rheumatoid Arthritis; OR (95% CI), odds ratio and 95% confidence interval, adjusted for ethnicity, formal education, age, sex, residential area, calculated by means of unconditional logistic regression to increase power; RA, rheumatoid arthritis; SE, shared epitope. **^a ^**HLA-DRB1*04:05 group was defined by the presence of either one or two copies of HLA-DRB1*04:05 allele. **^b^**Individuals with first allele of HLA-DRB1*04:05 and second allele of a non-HLA-DRB1*04:05 SE (that is, HLA-DRB1*04:05/non-HLA-DRB1*04:05 SE) were not included in the interaction analysis (cases, 13; control, 1). **^c^**Any non-HLA-DRB1*04:05 SE alleles group was defined by the presence of either one or two copies HLA-DRB1*01:01, *01:02, *01:07, *04:01, *04:04, *04:08, *04:10, *10:01, and *10:03 alleles.

The same pattern of interaction was observed when RF (IgM and IgG) was used for the subdivision of RA cases: *HLA-DRB1 *SE alleles were found to interact significantly with smoking only for RF-positive RA (for IgM RF, OR = 23.5; 95% CI, 9.5 to 57.8; AP = 0.7, 95% CI, 0.6 to 0.9; and for IgG RF, OR = 19.4; 95% CI, 7.9 to 47.8; AP = 0.7, 95% CI, 0.5 to 0.9), but not for the RF-negative RA subgroup (data not shown).

We also separately analyzed data only from the largest ethnic group in our study, the Malays. Smokers carrying SE alleles had a remarkably increased risk of ACPA-positive RA (OR = 32.0; 95% CI, 8.7 to 117.9; AP = 0.8; 95% CI, 0.5 to 1.1), compared with SE-negative never-smokers. The results for the two other major ethnic groups, Chinese and Indians, showed the same pattern but, because of the small number of observations, the precision was very low.

## Discussion

Two principal findings are presented in this case-control study. First, both smoking and the SE alleles were associated mainly with an increased risk of ACPA-positive RA. Second, we demonstrated a strong gene-environment interaction between smoking and SE alleles, which was restricted to only ACPA-positive RA in a multiethnic Malaysian population of Asian descent. Interestingly, this interaction seems to appear also between smoking and the SE allele HLA-DRB1*04:05, common in Asian but not among Caucasian subjects. Thus, our data extend previous results based on the Caucasian populations [6,7,10,11,13] to be observed also in an Asian population.

Our current study was designed as a case-control study with primarily early RA cases with the aim of investigating the impact of environmental, life-style, and genetic factors on the development of RA in an Asian population. The study was performed with the Swedish EIRA study as prototype [11] and has the advantage of being a large case-control study involving RA cases with short disease duration. A possible disadvantage with a case-control study using retrospectively collected data is the risk of recall bias, which occurs if the cases recall their previous exposure differently from the controls. Therefore, we included only cases with short disease duration to facilitate more-accurate information about their smoking habits before disease onset. Furthermore, in a previous report on smoking and the risk of RA, we performed a separate analysis on cases with symptom durations of less than 1 year to investigate possible recall bias [16]. In that analysis, ever-smoking was associated with an increased risk of ACPA-positive RA, which was in accordance with the results for which information from all cases was used.

In this report, we included both hospital- and population-based controls. Hospital controls might introduce selection bias if they do not reflect the smoking habits in the study base that generated the cases in our study. To elucidate this potential bias, we conducted separate analyses based on hospital and population controls, respectively. The results between these analyses were consistent, and thus we decided to merge the two control groups into one control group in the analyses to increase the power.

We previously demonstrated a profound interaction between smoking and SE alleles concerning risk of developing ACPA-positive RA in the Swedish EIRA population-based case-control study. We also provided evidence that cigarette smoking may induce citrullination in cells from the lungs [11]. Subsequent studies replicated and extended these findings, although mainly among Caucasians [5,6,8-10,19]. Similarly, smoking and SE alleles were also associated with the risk of developing RA, irrespective of RF or ACPA status in a Korean case-control study based on prevalent RA cases. However, interaction between smoking and SE alleles was statistically significant only for autoantibody-positive RA [4].

The genetic heterogeneity in our multiethnic study population is a major concern. We therefore performed stratified analyses by ethnicity, but the number of observations was too small to permit a meaningful analysis, except for the Malays. However, all the ethnic groups were showing similar trends toward the risk developing RA. Therefore, we decided to analyze them together to raise the statistical power for analyzing the exposures in question. As a result, we demonstrated a strong gene-environment interaction between smoking and SE alleles in ACPA-positive RA. These results are in accordance with those of other studies [10,11,13]. Nevertheless, these data are also in line with our previously reported hypothesis that cigarette smoking modulates the loss of tolerance to citrullinated autontigens occurring in SE-positive individuals [11].

Intriguingly, comparisons of populations with different genetic backgrounds but similar environmental exposure (smoking) can shed light on the general influence of non-genetic factors. Some SE alleles, like HLA-DRB1*04:05 and HLA-DRB1*10:01, are common in Asia [15] but not in Caucasian populations, in which instead HLA-DRB1*04:01 and HLA-DRB1*04:04 are common. An article recently published by our group demonstrated that, regardless of fine specificity, all *HLA-DRB1 *SE alleles strongly interacted with smoking in the development of ACPA-positive RA in the Swedish Caucasian population [7]. The HLA-DRB1*04:05 allele is the most common SE-encoding HLA-DRB1*04 subtype associated with RA reported in Asian populations, but is rare in Caucasian populations [15,20-22]. However, we are not aware of any published studies on interaction between smoking and distinct SE alleles in the development of RA defined by ACPA-subsets in multiethnic Asian populations. We observed that ever-smokers carrying the HLA-DRB1*04:05 allele had a 12.9-fold increased risk of ACPA-positive RA, and that signs of interaction were found between smoking and HLA-DRB1*04:05, although the interaction was not statistically significant (AP = 0.4; 95% CI, -0.1 to -0.9). Additional studies are needed for replication of these findings in other populations.

Several studies have reported positive interaction between SE and the cumulative dose of smoking and RA risk [9,19]. In this report, that kind of interaction analysis was not possible to perform because of small number of heavy smokers with SE alleles.

Finally, in the Swedish EIRA study, we found that smoking was responsible for approximately 35% of the ACPA-positive RA cases [23]. Although the female smoking prevalence was much lower in the present study, we estimated that smoking was responsible for approximately 8% of the ACPA-positive RA cases (1% for women, 45% for men). Because an increasing smoking trend is found in women in Asia, public health measures to prevent smoking are of importance.

## Conclusions

In conclusion, our findings indicate a gene-environment interaction between smoking and SE alleles in the development of ACPA-positive RA, but not in the development of ACPA-negative RA in a multiethnic population of Asian descent. This interaction also seems to apply between smoking and an *HLA-DRB1 SE *allele (HLA-DRB1*04:05) common in Asian populations but not in Caucasians. Our data are in accordance with previous findings among Caucasians and thus extend the basis for RA pathogenesis hypothesis involving genetic and environmental factors. Taken together, these findings may provide a new insight into the interplay between two major RA-susceptible risk factors (smoking and SE alleles) along one or more biologic pathways, which are common for the RA disease itself regardless of population origins.

## Abbreviations

ACPAs: anti-citrullinated protein antibodies; ACR: American College of Rheumatology; anti-CCP: anti-cyclic citrullinated peptide antibody; AP: attributable proportion; AU: arbitrary unit; CI: confidence interval; DNA: deoxyribonucleic acid; ELISA: enzyme-linked immunosorbent assay; *HLA-DRB1*: human leukocyte antigen-DR beta chain 1; IQR: interquartile range; MyEIRA: Malaysian Epidemiological Investigation of Rheumatoid Arthritis; OR: odds ratio; PCR-SSO: polymerase chain reaction-sequence-specific oligonucleotide; RA: rheumatoid arthritis; SAS: statistical analysis system; SE: shared epitope; Swedish EIRA: Swedish Epidemiological Investigation of Rheumatoid Arthritis.

## Competing interests

The authors declare that they have no competing interests.

## Authors' contributions

TCL and AY had full access to all of the data in this study and take responsibility for the integrity of the data and the accuracy of the data analysis. TCL and AY drafted the manuscript and performed statistical analysis and manuscript preparation. TCL, AY, SM, JSD, NAM, NAA, ANM, PTL, LK, LA, LP, and CB conceived the study and participated in the design of the study and manuscript editing. TCL, JSD, and LP performed serologic and molecular genetics assay. NAM and NAA contributed through the assessment of clinical aspects. TCL, AY, LP, and CB take responsibility for the acquisition of data and the analysis and interpretation of data. All authors were involved in drafting or revising the manuscript critically for important intellectual content, and all authors approved the final version to be published.
